# Differential Mitochondrial Adaptation in Primary Vascular Smooth Muscle Cells from a Diabetic Rat Model

**DOI:** 10.1155/2016/8524267

**Published:** 2016-01-11

**Authors:** Amy C. Keller, Leslie A. Knaub, P. Mason McClatchey, Chelsea A. Connon, Ron Bouchard, Matthew W. Miller, Kate E. Geary, Lori A. Walker, Dwight J. Klemm, Jane E. B. Reusch

**Affiliations:** ^1^Division of Endocrinology, University of Colorado Anschutz Medical Campus, Aurora, CO 80045, USA; ^2^Department of Medicine, Denver VA Medical Center, Denver, CO 80220, USA; ^3^Department of Bioengineering, University of Colorado Anschutz Medical Campus, Aurora, CO 80045, USA; ^4^Department of Cardiology, University of Colorado Anschutz Medical Campus, Aurora, CO 80045, USA; ^5^Division of Pulmonary Sciences and Critical Care Medicine, University of Colorado Anschutz Medical Campus, Aurora, CO 80045, USA; ^6^Center for Women's Health Research, University of Colorado School of Medicine, Aurora, CO 80045, USA

## Abstract

Diabetes affects more than 330 million people worldwide and causes elevated cardiovascular disease risk. Mitochondria are critical for vascular function, generate cellular reactive oxygen species (ROS), and are perturbed by diabetes, representing a novel target for therapeutics. We hypothesized that adaptive mitochondrial plasticity in response to nutrient stress would be impaired in diabetes cellular physiology via a nitric oxide synthase- (NOS-) mediated decrease in mitochondrial function. Primary smooth muscle cells (SMCs) from aorta of the nonobese, insulin resistant rat diabetes model Goto-Kakizaki (GK) and the Wistar control rat were exposed to high glucose (25 mM). At baseline, significantly greater nitric oxide evolution, ROS production, and respiratory control ratio (RCR) were observed in GK SMCs. Upon exposure to high glucose, expression of phosphorylated eNOS, uncoupled respiration, and expression of mitochondrial complexes I, II, III, and V were significantly decreased in GK SMCs (*p* < 0.05). Mitochondrial superoxide increased with high glucose in Wistar SMCs (*p* < 0.05) with no change in the GK beyond elevated baseline concentrations. Baseline comparisons show persistent metabolic perturbations in a diabetes phenotype. Overall, nutrient stress in GK SMCs caused a persistent decline in eNOS and mitochondrial function and disrupted mitochondrial plasticity, illustrating eNOS and mitochondria as potential therapeutic targets.

## 1. Introduction

Diabetes imparts staggering social and economic costs worldwide. It is known that people with diabetes have a 3–5-fold higher risk of cardiovascular disease (CVD) than the nondiabetic population [1]. Vascular remodeling, characterized by endothelial dysfunction and vascular stiffness and seen in the context of diabetes, hyperglycemia, and elevated oxidative stress, heralds CVD onset [2, 3]. Elucidating early cellular mechanistic pathology is critical to understanding disease progression.

Mitochondria have recently emerged as therapeutic targets in chronic diseases. They are critical signaling hubs in vascular processes such as the endothelial regulation of vasomotion, calcium signaling associated with vascular relaxation, smooth muscle cell proliferation, and apoptosis [4–6]. Excess cellular ROS in the vasculature may originate from dysfunctional mitochondria, and this excess ROS precedes vascular inflammation, vascular stiffness, and SMCs apoptosis [7–10]. Excess mitochondrial ROS is also associated with characteristics of type 2 diabetes such as hyperglycemia, decreased antioxidant defense, and insulin resistance [11–15]. In turn, hyperglycemia and insulin resistance also correlate with altered mitochondrial function, dynamics, and morphology [11, 15–20].

Nitric oxide (NO), produced by the enzyme nitric oxide synthase (NOS), regulates not only vascular relaxation [21], but also mitochondrial biogenesis through the activation of peroxisome proliferator-activated receptor *γ* coactivator 1*α* (PGC-1*α*) [22–24]. NOS dysfunction is a characteristic of both diabetes and CVD [25–28], and excess ROS contributes to uncoupled NOS (NOS uncoupled from NO production) in diabetes through the inactivation of cofactor tetrahydrobiopterin (BH_4_) [25, 29]. Uncoupled NOS is a known additional source of excess cellular superoxide [25]. Our laboratory has shown that disrupted NOS activity is also linked to suboptimal mitochondrial biogenesis signaling downstream [30].

Here, we aimed to characterize the role of NOS in mitochondrial plasticity in aortic primary smooth muscle cells (SMCs) from both control rats (Wistar) and a model of type 2 diabetes (Goto-Kakizaki, GK). We used primary SMCs from the GK model to investigate impaired mitochondrial plasticity based on our report of failed mitochondrial adaptation to exercise in vivo in this model [31]. We hypothesized that impaired NOS activity would result in dampened physiological responses to nutrient stress in GK SMCs as compared to those from Wistar.

## 2. Material and Methods

### 2.1. Materials

Dulbecco's Modified Eagles Medium (DMEM, 5 mM glucose, 25 mM glucose) and nonessential amino acids, trypsin, and mammalian protein extraction reagent (M-PER) were obtained from Thermo Scientific Hyclone (MA, USA), and dimethyl sulfoxide (DMSO), sodium chloride, sucrose, and bovine serum albumin were purchased from Fisher Scientific (PA, USA). Penicillin/streptomycin and FBS were procured from Gemini Bioproducts (CA, USA). Hank's Balanced Salt Solution (HBSS) was purchased from Corning Life Sciences (NY, USA). Collagenase, ethylenediaminetetraacetic acid (EDTA), ethylene glycol tetra-acetic acid (EGTA), sodium pyrophosphate, sodium orthovanadate, sodium fluoride, okadaic acid, 1% protease inhibitor cocktail, dithiothreitol, magnesium chloride, K-lactobionate, taurine, potassium phosphate, HEPES, digitonin, pyruvate, malic acid, glutamic acid, adenosine diphosphate (ADP), succinic acid, oligomycin, carbonyl cyanide 4-(trifluoromethoxy)phenylhydrazone (FCCP), antibody to *β*-actin (mouse), phenylephrine and acetylcholine, trypsin inhibitor, and cytochrome c were procured from Sigma-Aldrich (MO, USA). MitoTracker, MitoSOX, DAPI (4′,6-diamidino-2-phenylindole), dihydrochloride, and secondary detection antibodies Alexa Fluor 488 and Alexa Fluor 546 were purchased from Life Technologies (CA, USA). Antibodies to TOM20 (rabbit) and nitrotyrosine (mouse) were procured from Santa Cruz Biotechnology (TX, USA).

Antibodies: antibodies to adenosine monophosphate kinase (AMPK, 1 : 500 rabbit), phosphorylated AMPK (pAMPK, 1 : 1,000, rabbit, Thr172), autophagy-related protein 7 (Atg7, 1 : 500, rabbit), Beclin-1 (1 : 1,000, rabbit), light chain (LC3B-I/II, 1 : 1,000, rabbit), and anti-mouse (1 : 10,000) and anti-rabbit (1 : 10,000) IgG (AP-linked antibodies) were obtained from Cell Signaling (MA, USA). Antibodies to cytochrome c (1 : 1,000, mouse) and eNOS (1 : 200, mouse) and OPA1 (1 : 1,000, mouse) were from BD Biosciences (NJ, USA). Antibody to mitofusin-1 (Mfn1, 1 : 1,000, rabbit), mitofusin-2 (Mfn2, 1 : 500, mouse), uncoupling protein 3 (UCP3, 1 : 500, rabbit), and voltage dependent anion channel 1 (VDAC1, 1 : 1,000, rabbit) were from Abcam (Cambridge, UK). Antibody cocktail to representative subunits of mitochondrial oxidative phosphorylation (OxPhos) complexes I (subunit NDUFA9), II (subunit SDHA), III (subunit UQCRC2), IV (subunit IV), and V (subunit ATP5A) (1 : 1,000, mouse) were obtained from MitoSciences (OR, USA). Antibodies to phosphorylated eNOS (peNOS, 1 : 200, rabbit, S1177), citrate synthase (1 : 1,000, goat), PPAR*γ* coactivator 1 alpha (PGC-1*α*, 1 : 500, rabbit), and anti-goat IgG (1 : 10,000, AP-linked secondary antibody) were from Santa Cruz Biotechnology (TX, USA). MnSOD antibody (1 : 1,000, rabbit) was procured from EMD Millipore (MA, USA). The antibody to nucleoporin p62 (p62, 1 : 1,000, rabbit) was purchased from Sigma-Aldrich (MO, USA). Fis1 antibody (1 : 500, rabbit) was obtained from Imgenex (CO, USA). Immobilon-P PVDF membrane was from EMD Millipore (MA, USA). CDP-Star Reagent was obtained from New England BioLabs (MA, USA) and Life Technologies (CA, USA). Secondary antibodies (1 : 10,000, mouse, and 1 : 10,000, rabbit) for Western blot detection were purchased from Li-COR (NE, USA). Hoechst dye was obtained from Biotium (Hayward, CA).

### 2.2. Animals

The use of animals and experimental interventions received prior approval from the Institutional Animal Care and Use Committee at the Denver VA Medical Center. This study followed the principals of animal care as described by the National Institutes of Health. Primary aortic cells were harvested from male 22-week-old Wistar and GK rats (Taconic Biosciences, Inc., NY, USA, and Charles River Laboratories, MA, USA) as previously described [32].

### 2.3. Measurement of Contractile Function

Aortae were excised, placed in ice-cold physiological saline solution, debrided of loose fat and connective tissue, and prepared for measurement of isometric force as previously described [33–35]. The thoracic segment of aorta was dissected free from surrounding tissues and cut into rings of 2 mm in length. The preparation was then transferred into organ baths containing Krebs solution bubbled with a mixture of 95% O_2_ and 5% CO_2_. Each aortic ring was mounted between two L-shaped stainless steel hooks, one of which was connected to a force-displacement transducer (Grass Instruments Co., WI, USA). Krebs solution contained (mM): 119 NaCl, 4.7 KCl, 2.5 CaCl_2_, 1 MgCl_2_, 25 NaHCO_3_, 1.2 KH_2_PO_4_, and 11 D-glucose. Basal tension (500 mg) was applied to each ring, and all experiments were performed at 37°C. Tissues were contracted with 80 mmol/L KCl and then contracted with 2 *μ*M phenylephrine. Endothelial dependent relaxation was stimulated with 20 *μ*M acetylcholine. Data were collected using AcqKnowledge software.

### 2.4. Cell Isolation, Culture, and Maintenance

Aortae were excised, cleaned, and incubated in euglycemic media (DMEM 5 mM glucose supplemented with 10% FBS, 1% nonessential amino acids, and 1% penicillin/streptomycin) for 1-2 hours at 37°C, followed by an incubation in collagenase buffer (1 mg/mL of collagenase, 1 mg/mL of trypsin inhibitor, and 1 mg/mL of bovine serum albumin in euglycemic media) for 1 hour at 37°C while periodically vortexed. Aortae were finely cut into pieces (approximately 1–3 mm in length) in euglycemic media (20% FBS) and incubated at 37°C overnight. Experiments were conducted between passages 8 and 10, and cells were characterized using *α*-tubulin. Cells were maintained at 95% air and 5% CO_2_.

### 2.5. Cell Experimentation

SMCs were incubated in 0.1% serum starvation media for 45–48 hours (DMEM 5 mM glucose, 0.1% FBS, 1% nonessential amino acids, and 1% penicillin/streptomycin). SMCs were then preincubated for 30 minutes with normal glucose media NG (DMEM 5 mM glucose, 0.1% FBS, 1% nonessential amino acids, 1% penicillin/streptomycin, and 2% dimethyl sulfoxide (DMSO)) and then incubated for 1 and 4 hours with either NG or high glucose (HG, DMEM 25 mM glucose, recipe as described above). (We refer to normal glucose (NG, 5 mM) for 1 hour after media change as baseline and high glucose (HG, 25 mM) for either 1 or 4 hours as stress response.) Glucose or vehicle (PBS) was directly added into media for 1- and 4-hour incubations for SMCs used for flow cytometry and the ATP assay, and microscopy time course experiments used serum starvation media described above with 25 mM glucose. All experiments were conducted in triplicate.

### 2.6. Respiration

Mitochondrial respiration was measured using Oroboros Oxygraph-2K (O2k, OROBOROS INSTRUMENTS Corp., Innsbruck, Austria) according to modifications from previously described protocols [31, 36, 37]. Briefly, SMCs were trypsinized using 0.25% trypsin/EDTA and washed and spun (3 minutes at 1,000 g) once each with phosphate buffer saline (PBS) and MiR05 respiration buffer (0.5 mM EGTA, 3 mM magnesium chloride, 60 mM K-lactobionate, 20 mM taurine, 10 mM potassium phosphate, 20 mM HEPES, 110 mM sucrose, and 1 g/L fatty acid free bovine serum albumin). Following the final spin, SMCs were resuspended in MiR06 respiration buffer (MiR05 with 280 IU/mL catalase) and counted using a hemocytometer. Between 0.5 × 10^6^ and 1 × 10^6^ SMCs were placed into a 2 mL chamber of the O2k and permeabilized with 3 *μ*g of digitonin. After respiration rates stabilized, substrates and inhibitors were added to assess respiration rates. Rates were measured following the addition of 5 mM pyruvate, 2 mM malate, and 10 mM glutamate (state 2 PMG), PMG with 2 mM adenosine diphosphate (ADP) (state 3 PMG), PMG, ADP, and 6 mM succinate (state 3 PMGS), and 2 *μ*g/mL oligomycin (state 4 PMGS), and 0.5 *μ*M of carbonyl cyanide 4-(trifluoromethoxy)phenylhydrazone (FCCP) was added incrementally until maximal uncoupling (uncoupled state). Cytochrome c (10 *μ*M) was used to determine mitochondrial membrane damage. There were no significant differences in oxygen consumption following cytochrome c addition, indicating intact mitochondrial membranes. Oxygen and all substrates were calibrated to be at saturating concentrations in the chambers with no possibility of rate limitations. The respiration technique described above has been optimized for SMCs. Upon completion of the experiment, SMCs were recounted from the O2k chambers and respiration rates normalized to cell count. An area of consistent respiration rate of 3 minutes or longer was representative of the various states. Respiration control ratios (RCR) were calculated as a ratio of state 3 PMGS/state 4 PMGS.

### 2.7. ATP Assay

ATP concentrations were measured in cells using the Fluorometric ATP Assay Kit (#ab83355) from Abcam, Cambridge, United Kingdom. Briefly, following the experiment, cells were harvested, counted, and protein-precipitated according to manufacturer instructions. After adjusting the samples' pH to between 6.5 and 8, samples were incubated with the kit reagents according to the fluorometric protocol, and ATP concentrations were normalized to 1 × 10^6^ cells.

### 2.8. Western Blotting

SMCs were harvested after incubations and protein was measured using Western blotting as previously described [31]. SMCs were harvested after incubations in 4°C mammalian lysis buffer (MPER with 150 mM sodium chloride, 1 mM of EDTA, 1 mM EGTA, 5 mM sodium pyrophosphate, 1 mM sodium orthovanadate, 20 mM sodium fluoride, 500 nM okadaic acid, and 1% protease inhibitor cocktail). After sonication at 4°C, cell lysates were centrifuged at 18,000 ×g at 4°C for 10 min, and the protein concentration of the supernatant was analyzed by Bradford protein assay. Protein samples (15 *μ*g to 40 *μ*g) in Laemmli sample buffer (boiled with 100 mM dithiothreitol) were run on SDS-12% polyacrylamide gels. The resolved proteins were electrophoretically transferred to PVDF membranes, and equivalence of protein loading was initially assessed by staining of membrane-bound proteins by Ponceau S stain. Blots were probed using specific primary antibodies of interest (overnight at 4°C) and followed by either alkaline phosphatase- (AP-) linked or fluorescent secondary (1 : 1000, 1 hr at room temperature). Proteins were detected by chemiluminescence using CDP-Star Reagent on film or Li-COR (Odyssey CLX) Western blot scanner, and densitometric analysis was performed using either Quantity One or Image Studio v3.1. All data is normalized to *β*-actin protein expression. We detected eNOS monomers and dimers using a 7.5% SDS-page gel transferred to PVDF membrane at 4°C under nonreducing conditions, as described previously with modifications [38]. Samples were prepared with Laemmli buffer without reducing agent and not boiled. Detection and analysis were done as described above.

### 2.9. Mitochondrial Isolation and Enzyme Activity Assay

Treated SMCs were harvested in 10 mM Tris (pH 7.6). Isolated mitochondria were obtained by homogenization (10 passes through a syringe with a 25-26 G needle) followed by centrifugation through a 1.5 M sucrose gradient. Final mitochondrial isolates were subjected to 3 freeze/thaw cycles and activity of respiratory chain enzyme complexes I + III and II + III and citrate synthase (CS) was measured spectrophotometrically as previously described [39] on a BioTek Synergy (VT, USA) H1 microplate reader. Enzyme activities were normalized to protein (using the Bradford method).

### 2.10. Nitric Oxide Synthase Activity Assay

SMCs were seeded at 40,000 to 60,000 cells per well in a 96-well plate. Following a 48-hour serum starvation, SMCs were treated as described and assayed for nitric oxide (NO) evolution according to manufacturer's instructions (Nitric Oxide Synthase Detection System, Sigma-Aldrich, MO, USA, #FCANOS1). Live cells were stained using Hoechst's dye at 0.5 *μ*g/mL for 20 minutes at 37°C prior to reading fluorescence and data expressed as relative fluorescence units (RFU) of NO concentration normalized to live cells.

### 2.11. Amplex Red Assay

SMCs between passages 3 and 7 were cultured in 5 mM glucose DMEM. Following 48-hour serum deprivation, H_2_O_2_ accumulation was measured using an Amplex Red Hydrogen Peroxide/Peroxidase Assay Kit (#A22188) Invitrogen, CA, USA. The standard curve was done in live cells to control for quenching. Data are presented in *μ*M equivalents of H_2_O_2_.

### 2.12. Flow Cytometry

Treated SMCs were trypsinized, and 150,000 cells were spun for 3 minutes at 1,000 g. Cells were resuspended in MitoTracker (final concentration of 9 nM in HBSS) and incubated at 37°C in darkness for 20 minutes. Cells were spun and resuspended in MitoSOX (final concentration of 5 *μ*M in HBSS) and incubated for 37°C in dark conditions for 15 minutes. DAPI was added for live cell gating, and Beckman Coulter Gallios flow cytometer was used (CA, USA, University of Colorado Cancer Center Flow Core).

### 2.13. Microscopy and Immunohistochemistry

SMCs were cultured on a glass coverslip, exposed to HG media for 1 or 4 hours, and compared to a NG media baseline. Following glucose exposure, samples were fixed by rinsing once with PBS prewarmed to 37°C and incubated at 37°C for 15 minutes in 37°C 4% paraformaldehyde in PBS. After fixation, samples were then incubated for 15 minutes in 50 mM NH_4_Cl in PBS. Finally, samples were stored in PBS at 4°C until staining. Mitochondrial and nitrotyrosine staining were completed by adding TOM20 (1 : 400) and nitrotyrosine (1 : 400) antibodies, respectively, followed by secondary detection antibodies Alexa Fluor 488 (1 : 2,000) and Alexa Fluor 564 (1 : 1,000). DAPI (1 : 1,000) was used to visualize the nuclei. Fixed cells were imaged using an Olympus confocal FV1000 FCS/RICS microscope. Experiments were repeated in triplicate (*n* = 3) on separate days, and three plates were tested at each time point in each experiment, for a total of *n* = 9 plates per time point. In addition, three fields of view were imaged and analyzed on each plate, for a total of *n* = 27 fields of view imaged per experimental time point. Raw images acquired from confocal microscopy were imported into Matlab in 24-bit TIFF format and converted into binary images using a program designed by one of the authors (PMM). To assess differences in mitochondrial morphology, the total mitochondrial network edge length within the field of view was divided by the total number of mitochondria to give an average perimeter per mitochondrion. To assess differences in mitochondrial content, the total area of the mitochondrial network was divided by the total area of cytoplasm to yield the fraction of the cytoplasm filled by mitochondria. Mitochondrial content was found to be correlated with the inverse of average cell size within the field of view in all cell populations at all time points. The fraction of cytoplasm filled by mitochondria was therefore normalized to median cell size using a linear regression in order to prevent aberrant detection of changes in mitochondrial content due to random variation in the size of cells measured between time points. No correlation was observed between average perimeter per mitochondrion and cell size. In addition to average perimeter per mitochondrion and fraction of cytoplasm filled, total network edge length, total network area, total cytoplasm area, total number of nuclei, average network area, and average cytoplasm area per cell were recorded for each field of view. Images which were registered at greater than two standard deviations from the mean value for their experimental group in any of these parameters were discarded as outliers. The mean and standard error of each group were computed from the remaining images.

### 2.14. Statistics

Either two-tailed Student's *t*-test or one-way ANOVA was used for data analysis. A *p* value of less than 0.05 was used as the cutoff for statistical significance in all tests.

## 3. Results

### 3.1. Contractility Impairments in GK Rat Aorta

**Figure 1:**
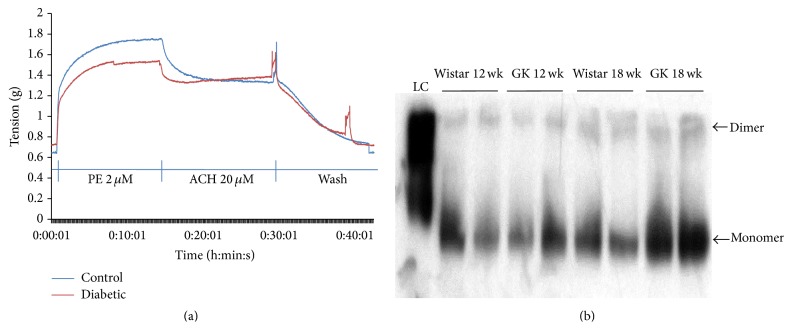
(a) Contractile responses of Wistar (control) and GK (diabetic) aorta smooth muscle. (b) Dimer (coupled) and monomer (uncoupled) eNOS in aorta of 18-week-old Wistar and GK animals. 15–30 *μ*g of protein was loaded onto a 7.5% SDS-page gel and transferred to PVDF membrane at 4°C under nonreducing conditions.

In the representative tracing, phenylephrine (2 *μ*M) evoked a greater contractile response in Wistar aorta than the GK aorta (Figure 1(a)), and addition of acetylcholine (20 mM) resulted in a greater relaxation response in the Wistar aorta than the GK aorta (Figure 1(a)). These differences strongly suggest impaired vascular function in the GK rat.

### 3.2. NOS Expression

In the representative blot pictured, dimer eNOS is visible at 8.09% higher concentrations in aorta from the 18-week-old GK as compared to the Wistar rats (Figure 1(b)), and 40% higher concentrations of the monomer of eNOS are seen in GK aorta (Figure 1(b)).

### 3.3. Baseline Mitochondrial Respiration Is Elevated in GK SMCs

The GK SMCs had significantly elevated state 3 respiration rates as well as respiratory control ratio (RCR, state 3 PMGS: state 4 PMGS), an indication of respiration efficiency [40, 41], at baseline as compared to Wistar SMCs (*p* < 0.05, Table 1). Additionally, ATP concentrations were nonsignificantly decreased in the GK SMCs (Table 1). To further characterize mitochondrial function, the activity of complexes I + III and II + III and citrate synthase was measured. Activity of complexes I + III in GK SMCs at baseline was nonsignificantly less as compared to Wistar SMCs (*p* = 0.088, data not shown).

### 3.4. Mitochondrial Biogenesis and NOS Signaling in GK SMCs

**Figure 2:**
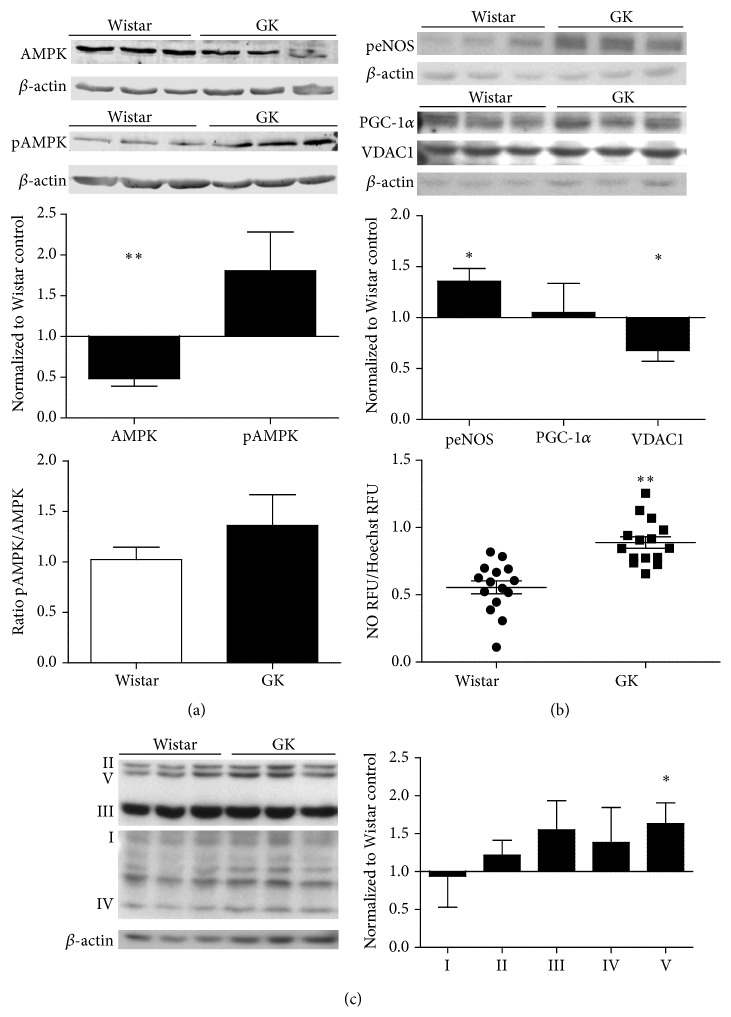
(a) Baseline comparisons of AMPK (*n* = 3), pAMPK (*n* = 3), and AMPK specific activity (pAMPK/AMPK), signaling upstream of mitochondria (*n* = 3) and resultant NO evolution (*n* = 4) (b), and mitochondrial complex differences (*n* = 3) (c) in Wistar and GK SMCs, passages 8–10. Signaling protein expression was measured with Western blot, 15–30 *μ*g protein on an SDS-page gel, and data are normalized to *β*-actin and expressed as mean fold change from Wistar SMCs + SEM. Significance measured by Student's *t*-test, ^*∗*^ *p* < 0.05. NO (*n* = 3) is expressed as relative fluorescence units (RFU) normalized to RFU of Hoechst live cell staining for cell number. ^*∗*^ *p* < 0.05, ^*∗∗*^ *p* < 0.01 as measured by Student's *t*-test.

AMPK expression at baseline was significantly less in GK SMCs as compared to Wistar cells (*p* < 0.01, Figure 2(a)). NO, as a measurement of NOS activity, was significantly elevated in GK SMCs (*p* < 0.001, Figure 2(b)). Phosphorylated eNOS protein expression was significantly elevated in GK SMCs (*p* < 0.05, Figure 2(b)). Unexpectedly, we observed no change in PGC-1*α*, suggesting a disconnect between eNOS activation and PGC-1*α* regulation (Figure 2(b)). Mitochondrial complexes II–V (I (subunit NDUFA9), II (subunit SDHA), III (subunit UQCRC2), IV (subunit IV), and V (subunit ATP5A)) were elevated in GK as compared to Wistar SMCs, yet only complex V differed significantly (*p* < 0.05, Figure 2(c)).

### 3.5. Unstimulated GK SMCs Produced Elevated ROS as Compared with Wistar SMCs

**Figure 3:**
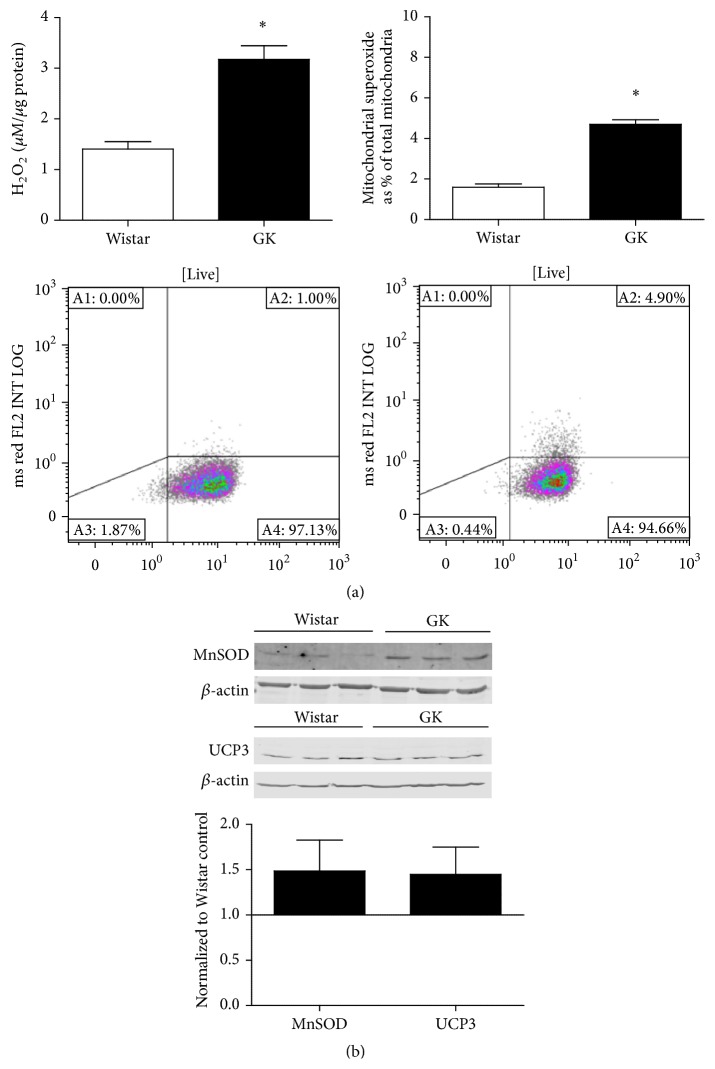
(a) Baseline reactive oxygen species differences between Wistar and GK SMCs in NG (5 mM). Hydrogen peroxide was measured by Amplex Red (*n* = 3), and superoxide was detected as a percentage of total mitochondria using MitoTracker and MitoSOX fluorescence on live cells with flow cytometry (*n* = 3). Representative flow cytometry figures of Wistar SMCs (left) and GK SMCs (right) are presented. (b) Mitochondrial antioxidant status was measured with Western blot, 15–30 *μ*g protein on an SDS-page gel, and data are normalized to *β*-actin and expressed as mean fold change from Wistar SMCs + SEM. Significance measured by Student's *t*-test, ^*∗*^ *p* < 0.05.

Baseline concentrations of H_2_O_2_ and superoxide relative to mitochondrial content revealed significantly elevated ROS in the GK cells as compared to the Wistar cells (*p* < 0.05, Figures 3(a) and 3(b)). No differences were seen in MnSOD or UCP3 protein expression (Figure 3(b)), indicators of endogenous antioxidant defenses.

### 3.6. Mitochondrial Morphology Differed between SMCs at Baseline

**Figure 4:**
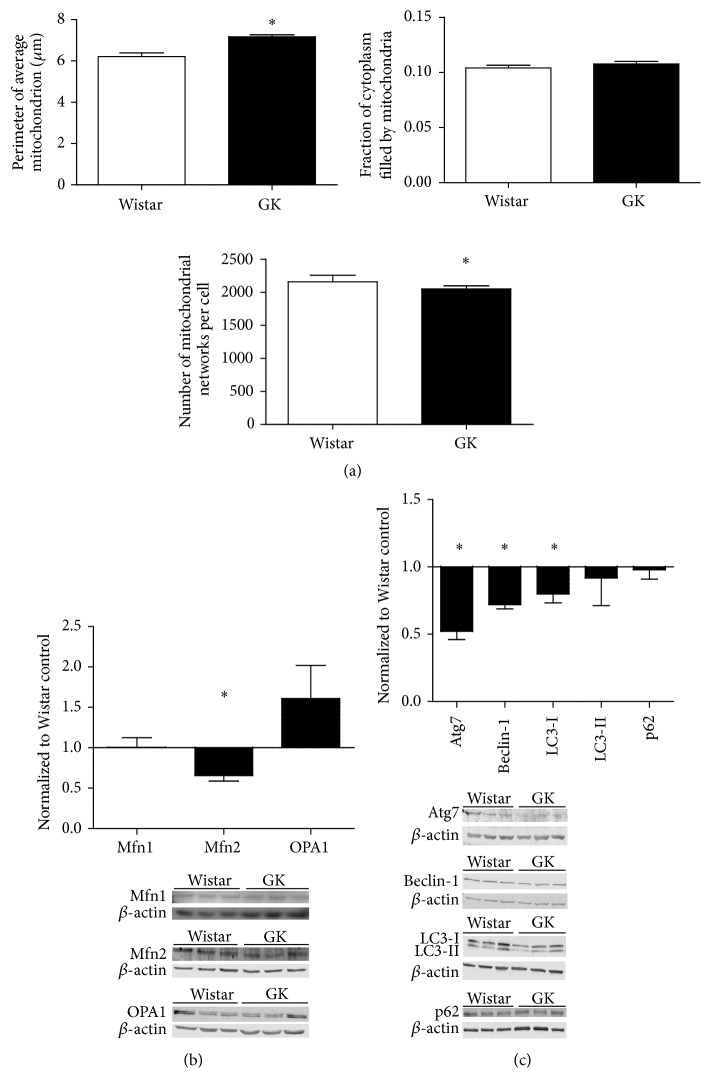
(a) Assessment of baseline mitochondrial morphology. Baseline perimeter per mitochondrion, total mitochondria fill of cytoplasm, and number of mitochondrial networks per cell were measured in fixed Wistar (*n* = 3) and GK (*n* = 3) SMCs using TOM 20 (mitochondria) and nitrotyrosine (cytoplasm). (b) Baseline comparison of mitochondrial dynamics and autophagy regulators (c) in Wistar (*n* = 3) and GK (*n* = 3) SMCs, passages 8–10. Signaling protein expression was measured with Western blot, 15–30 *μ*g protein on an SDS-page gel, and data are normalized to *β*-actin and expressed as mean fold change from Wistar cells + SEM. Significance measured by Student's *t*-test, ^*∗*^ *p* < 0.05.

Perimeter per mitochondrion was significantly elevated in the GK SMCs at baseline as compared to Wistar SMCs (*p* < 0.05, Figure 4(a)). In accord with this finding, the mean number of mitochondrial bodies per cell was significantly higher in the Wistar SMCs at baseline as compared to the GK SMCs (*p* < 0.05, Figure 4(a)). Fusion markers included OPA1, mitofusin-1 (Mfn1), and mitofusin-2 (Mfn2); fission was examined using Fis1. Expression of fusion molecule Mfn2 was significantly reduced in GK cells as compared to the Wistar SMCs at baseline (*p* < 0.05, Figure 4(b)). In comparison to the Wistar, autophagy targets Atg7, Beclin-1, and LC3-1 were significantly less in GK SMCs (*p* < 0.05, Figure 4(c)).

### 3.7. Mitochondrial Respiration in the Context of High Glucose Stress

The uncoupled state respiration rate in GK SMCs was significantly decreased following exposure to glucose stress (*p* < 0.05, Table 2), and respiration states 2 and 3 (PMG) showed a nonsignificant decline (*p* = 0.05 for both, Table 2). ATP concentrations were not significantly different following HG exposure (Table 2). No differences were observed in the enzyme activity of mitochondrial complexes I + III and II + III or citrate synthase in Wistar and GK cells in response to high glucose stress (data not shown).

### 3.8. Signaling Upstream of Mitochondrial Biogenesis and Function Is Disrupted in GK SMCs following Glucose Mediated Stress

**Figure 5:**
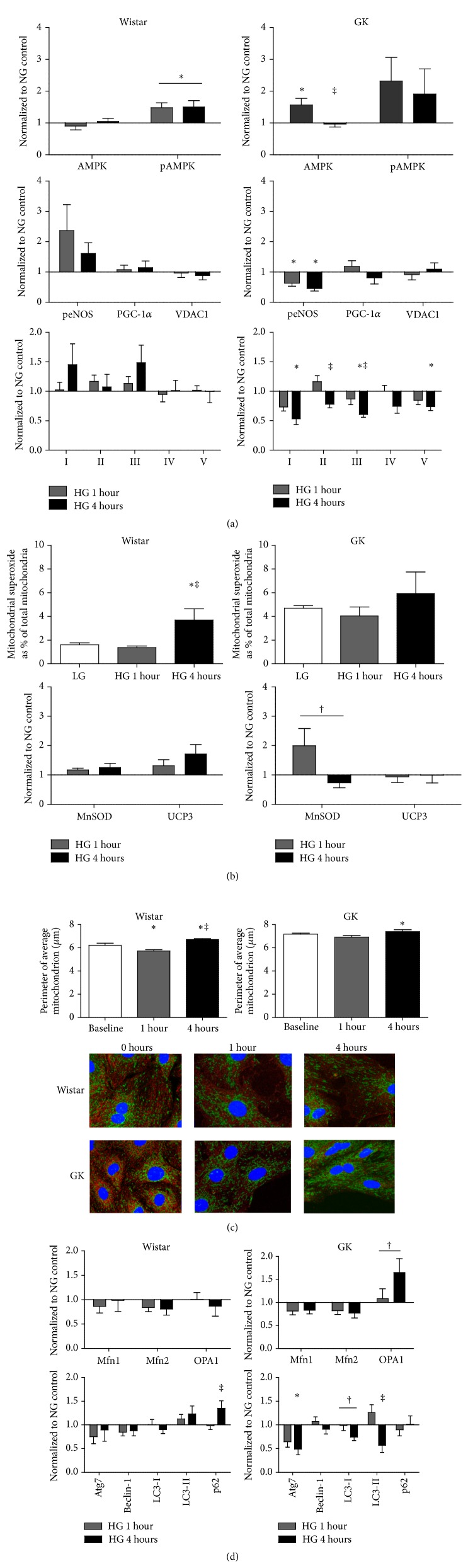
(a) Time course comparisons of AMPK (*n* = 3), pAMPK (*n* = 4), peNOS (*n* = 3), PGC-1*α* (*n* = 3), and VDAC1 (*n* = 3), and mitochondrial complex expression in Wistar and GK SMCs, passages 8–10. (b) Superoxide (flow cytometry, *n* = 3) and antioxidant status (Western blot, MnSOD *n* = 4, UCP3 *n* = 3) differences between Wistar and GK SMCs in NG (5 mM) or HG (25 mM). Protein expression was measured using Western blot, 15–30 *μ*g protein on an SDS-page gel, and data are normalized to *β*-actin and expressed as mean fold change from NG + SEM. (c) Assessment of mitochondrial morphology (*n* = 3) during HG (25 mM) time course. Representative photographs of fixed SMCs using TOM 20 (green) and nitrotyrosine (red) are shown. (d) Time course mitochondrial dynamic (*n* = 4) and autophagy (*n* = 3) differences in Wistar and GK SMCs, passages 8–10. Protein expression was measured using Western blot, 15–30 *μ*g protein on an SDS-page gel, and data are normalized to *β*-actin and expressed as mean fold change from NG + SEM. Significance measured by one-way ANOVA, *p* < 0.05 (*∗* compared to NG, ‡ compared to 1-hour HG), ^†^ *p* < 0.1.

There was a significant effect on pAMPK in Wistar and AMPK in GK SMCs with HG incubation (*p* < 0.05, Figure 5(a)). Additionally, phosphorylated eNOS was significantly reduced in GK SMCs after 1 hour and 4 hours of high glucose as compared to normal glucose (*p* < 0.05), with no change in Wistar SMCs (Figure 5(a)). In the GK SMCs, complexes I, II, III, and V were significantly decreased in response to 4 hours of HG treatment (*p* < 0.05 for all, Figure 5(a)).

### 3.9. ROS Production in GK and Wistar SMCs Was Differently Affected by Glucose Stress

Following 4 hours of HG incubation, mitochondrial superoxide production was significantly increased in the Wistar cells (*p* < 0.05, Figure 5(b)) whereas no changes were observed in GK cells (Figure 5(b)). UCP3 expression in the Wistar SMCs showed a nonsignificant increase following HG treatment (*p* = 0.1, Figure 5(b)), and MnSOD expression in the GK SMCs showed a nonsignificant treatment effect towards decreased expression with 4 hours of HG exposure (*p* = 0.07, Figure 5(b)).

### 3.10. GK SMCs Display Altered Mitochondrial Dynamics in Response to High Glucose Stress

At all time points measured, the average perimeter per mitochondrion among GK SMCs was significantly greater than in Wistar SMCs (*p* < 0.001, Figure 5(c)). The final average perimeter per mitochondrion in the Wistar SMCs exposed to 4 hours of glucose stress was significantly greater than at baseline (*p* < 0.05, Figure 5(c)). The final average perimeter per mitochondrion in the GK 4-hour group was also significantly greater than baseline (*p* < 0.05, Figure 5(c)). The fraction of cytoplasm filled by mitochondria significantly increased from 1 to 4 hours in GK cells (*p* < 0.05, data not shown). There was a nonsignificant increase of OPA1 protein expression following 1 and 4 hours of HG treatment in the GK SMCs (*p* = 0.06, Figure 5(d)). Following a 4-hour exposure to HG, Wistar SMCs showed a significant increase in p62 protein expression as compared to 1 hour of exposure (*p* < 0.05, Figure 5(d)); in response to HG, GK SMCs had a significant decline in protein expression of Atg7 and LC3-II (*p* < 0.05, Figure 5(d)) and nonsignificant decrease in LC3-1 protein expression (*p* = 0.08, Figure 5(d)).

## 4. Discussion

**Figure 6:**
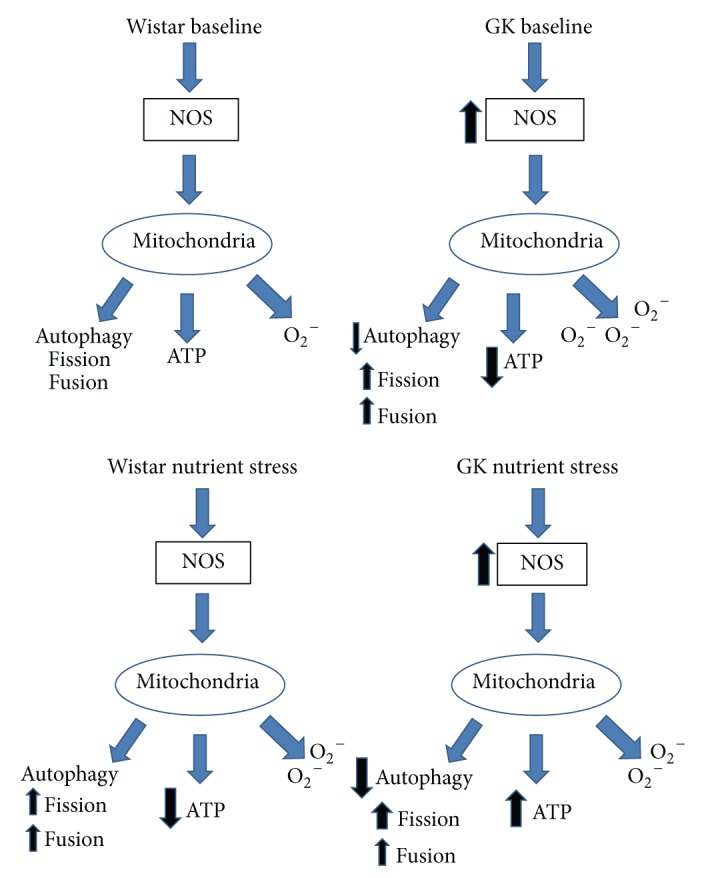
Data summary of cellular metabolic processes. Arrows indicate differences as compared with Wistar SMCs at baseline.

Two-thirds of individuals with type 2 diabetes die due to cardiovascular disease (CVD), despite control of hyperglycemia and traditional CVD risk factors [1, 42]. In order to address this 3–5-fold excess risk, new therapeutic targets must be identified. The current work further builds upon our previous studies by characterizing alterations in mitochondrial homeostasis in primary SMCs from control (Wistar) and diabetes (GK) rat vasculature in order to more specifically examine the mitochondrial functional implications of our preceding reports. We observe differential mitochondrial and NOS activity, cellular signaling upstream of mitochondrial biogenesis and function, elevated baseline ROS production, and most notably a pattern of different mitochondrial plasticity and ROS in response to glucose induced metabolic stress in GK SMCs ex vivo as compared to Wistar SMCs (Figure 6). Thus, primary GK and Wistar SMCs illustrate persistent cellular mitochondrial differences in culture. One noted observation was the altered expression of mitochondrial complexes in the GK SMCs independent of parallel differences in PGC-1*α*. This may reflect the activation of alternate pathways leading to mitochondrial biogenesis without the activity of PGC-1*α*, particularly in states of environmental stress, nutrient excess, or pathology, as described in previous studies utilizing excess pyruvate, a peroxisome-deficient cellular model, and an exercised* ob/ob* mouse model of diabetes [43–45].

SMCs activation is characterized by increases in oxidative phosphorylation [6]. We assessed mitochondrial respiration in permeabilized cells, which permit evaluation of mitochondrial function separate from intracellular machinery and neutralize the impact of intracellular fuel partitioning [40]. Unexpectedly, GK SMCs cultured in normal glucose displayed greater state 3 and RCR measures of oxidative function compared with Wistar SMCs. Along with elevated baseline ROS in the GK SMCs, these respiratory changes are consistent with the elevated respiration, greater ATP production, and subsequent excess mitochondrial ROS seen in vascular dysfunction [16, 18]. Following exposure to high glucose, we observed decreased states 2 and 3 and uncoupled respiration states in GK SMCs along with a significant increase in perimeter per mitochondria and content. This suggests that baseline mitochondrial functional differences between Wistar and GK are due to altered dynamics, not mass, as mass is increasing in both cell types with nutrient stress. Also, ATP production was not significantly affected by HG exposure, suggesting that the basal levels are stable in these cells in culture.

Interestingly, although NO concentration, a measurement of NOS activity, was elevated in the GK SMCs at baseline, NO concentrations remained unaffected upon treatment exposure in Wistar and GK SMCs despite a significant decline in peNOS. This suggests that although eNOS signaling upstream of mitochondrial function is decreased in the glucose stressed GK SMCs, any resultant effect on NO production may have been overcome in 4 hours. Also, our protein expression measurements represent a snapshot of regulation and may not capture acute signaling or cellular responses. Ultimately, these data support our hypothesis and suggest NOS as a therapeutic target.

There is significant support in the literature for the hypothesis that excess mitochondrial ROS production plays a causal role in diabetic microvascular complications [15]. Nutrient stress derived from both glucose and lipids has been postulated to increase mitochondrial ROS production, presumed secondary to excess nutrient flux through the electron transport chain [11, 15, 46, 47]. We observed significantly elevated baseline ROS, H_2_O_2_ production, and mitochondrial superoxide in the GK SMCs, indicating innate differences in mitochondrial and total cellular ROS in the GK as compared to the Wistar SMCs, consistent with previous reports in diabetes and endothelial cells [48–53]. In contrast, GK SMCs did not demonstrate the expected increase in ROS generation when exposed to HG. Consistent with this, GK SMCs exposed to HG decreased expression of mitochondrial complexes I, II, III, and V and mitochondrial respiration. This in vitro finding aligns with our recent report of failed adaptation to an exercise stimulus in the GK aorta [31].

Baseline GK SMCs are metabolically active with activation of mitochondrial biogenesis signaling, mitochondrial ROS production, fusion, and respiration. This increased respiration is different than the general decrease in mitochondrial function observed in skeletal muscle in diabetes [54, 55]. Our finding that oxidative stress and oxidative phosphorylation are elevated in normal glucose media and diminished in high glucose media is consistent with a model where the hyperglycemic state drives lessened mitochondrial function. Importantly, our observation that nutrient stress results in decreased mitochondrial biogenesis signaling suggests that acute metabolic stress, not intrinsic mitochondrial dysfunction, is responsible for the differential adaptation and decreased mitochondrial function. This differential adaptation is reminiscent of our previously reported decrease in mitochondrial respiration and biogenesis signaling observed with exercise mediated stress in diabetes and with L-NAME, an inhibitor of NOS [30, 31, 56].

Alterations in mitochondrial network dynamics have been reported in diabetes [17, 47]. Other studies observe that dysregulated mitochondrial fusion, seen across animal models of cardiovascular disease, is central to this disease progression [6, 17, 20, 57]. Our observation of greater perimeter per mitochondrion is consistent with greater fusion, which aligns with the observed elevation in respiration in the GK SMCs as compared with the Wistar SMCs. We also observed decreased expression of autophagy markers in GK SMCs exposed to nutrient stress, implying that the protective aspects of autophagy may be aberrant in diabetes [58].

The literature supports that diabetes decreases mitochondrial function and increases ROS [17, 31, 47, 54–56]. In contrast, our data indicate that primary SMCs from the diabetes rats have greater mitochondrial content and respiration, NO, eNOS, and cytosolic and mitochondrial ROS and that the decline in mitochondrial function requires exposure to nutrient stress (Figure 6). Altered mitochondrial plasticity in response to excess glucose may be one mechanism underlying vascular smooth muscle cell mitochondrial dysfunction and ROS production in vivo. In conclusion, these data endorse the importance of mitochondrial dysregulation in diabetic vascular disease and suggest a potential role for metabolic stress in the contractile dysfunction (stiffness) observed in diabetes.

## Supplementary Material

The supplementary material shows representative blots in support of Figures 5A, 5B, and 5D. Material and methods for these data are described in the main manuscript.

## Figures and Tables

**Table 1:** Mitochondrial respiration and ATP concentrations as measured with O2k from Oroboros and ATP fluorometric assay. Respiration baseline comparisons are between Wistar (*n* = 6) and GK (*n* = 3) SMCs in NG (5 mM), Wistar, or GK SMCs, passages 8–10. ATP concentrations (*µ*M/1 × 10^6^ cells) are between Wistar (*n* = 8) and GK (*n* = 8). Respiration was measured as oxygen flux (pmol second^−1^ million cells^−1^) ± standard error of the mean (SEM). ^*∗*^ *p* < 0.05 as measured by Student's *t*-test.

Respiration states	Wistar	GK
S2 (PMG)	18.35 ± 3.97	29.79 ± 5.38
S3 (PMG)	75.99 ± 19.04	153.88 ± 23.82^*∗*^
S3 (PMG/S)	187.82 ± 40.96	241.30 ± 36.46
S4 (PMG/S)	54.88 ± 8.33	52.52 ± 4.56
Uncoupled	262.98 ± 46.06	375.15 ± 20.63
RCR	3.35 ± 0.29	4.56 ± 0.45^*∗*^
ATP	8.64 ± 1.91	7.14 ± 1.08

**(a):** 

Respiration states: Wistar	NG	HG
S2 (PMG)	18.35 ± 3.97	8.61 ± 2.37
S3 (PMG)	75.98 ± 19.04	51.73 ± 12.93
S3 (PMG/S)	187.82 ± 40.96	131.07 ± 9.19
S4 (PMG/S)	54.88 ± 8.33	34.34 ± 1.13
Uncoupled	262.98 ± 46.06	166.38 ± 16.48
RCR	3.35 ± 0.29	3.44 ± 0.21
ATP	8.64 ± 1.91	6.97 ± 1.34

**(b):** 

Respiration states: GK	NG	HG
S2 (PMG)	29.79 ± 5.38	11.96 ± 1.49^†^
S3 (PMG)	153.88 ± 23.82	90.41 ± 13.78^†^
S3 (PMG/S)	241.30 ± 36.46	197.96 ± 20.64
S4 (PMG/S)	52.52 ± 4.56	46.17 ± 3.34
Uncoupled	375.15 ± 20.63	246.70 ± 22.60^*∗*^
RCR	4.56 ± 0.45	4.37 ± 0.11
ATP	7.14 ± 1.08	8.87 ± 1.90

## Abbreviations

AMPK: Adenosine monophosphate kinase
CVD: Cardiovascular disease
eNOS: Endothelial nitric oxide synthase
GK: Goto-Kakizaki
HG: High glucose
M-PER: Mammalian protein extraction reagent
NG: Normal glucose
NO: Nitric oxide
NOS: Nitric oxide synthase
PGC-1*α*: Peroxisome proliferator-activated receptor *γ* coactivator 1*α*
ROS: Reactive oxygen species
SMCs: Smooth muscle cells.
