# Supplementary material for: Differential Mitochondrial Adaptation in Primary Vascular Smooth Muscle Cells from a Diabetic Rat Model
Source: Oxid Med Cell Longev. 2016 Jan 11;2016:8524267. doi: 10.1155/2016/8524267 (PMC4737048; doi:10.1155/2016/8524267)
Supplement: Supplementary file 1 — The supplementary material shows representative blots in support of Figures 5A, 5B, and 5D. Material and methods for these data are described in the main manuscript. [file 8524267.f1.pptx]

## Slide 1
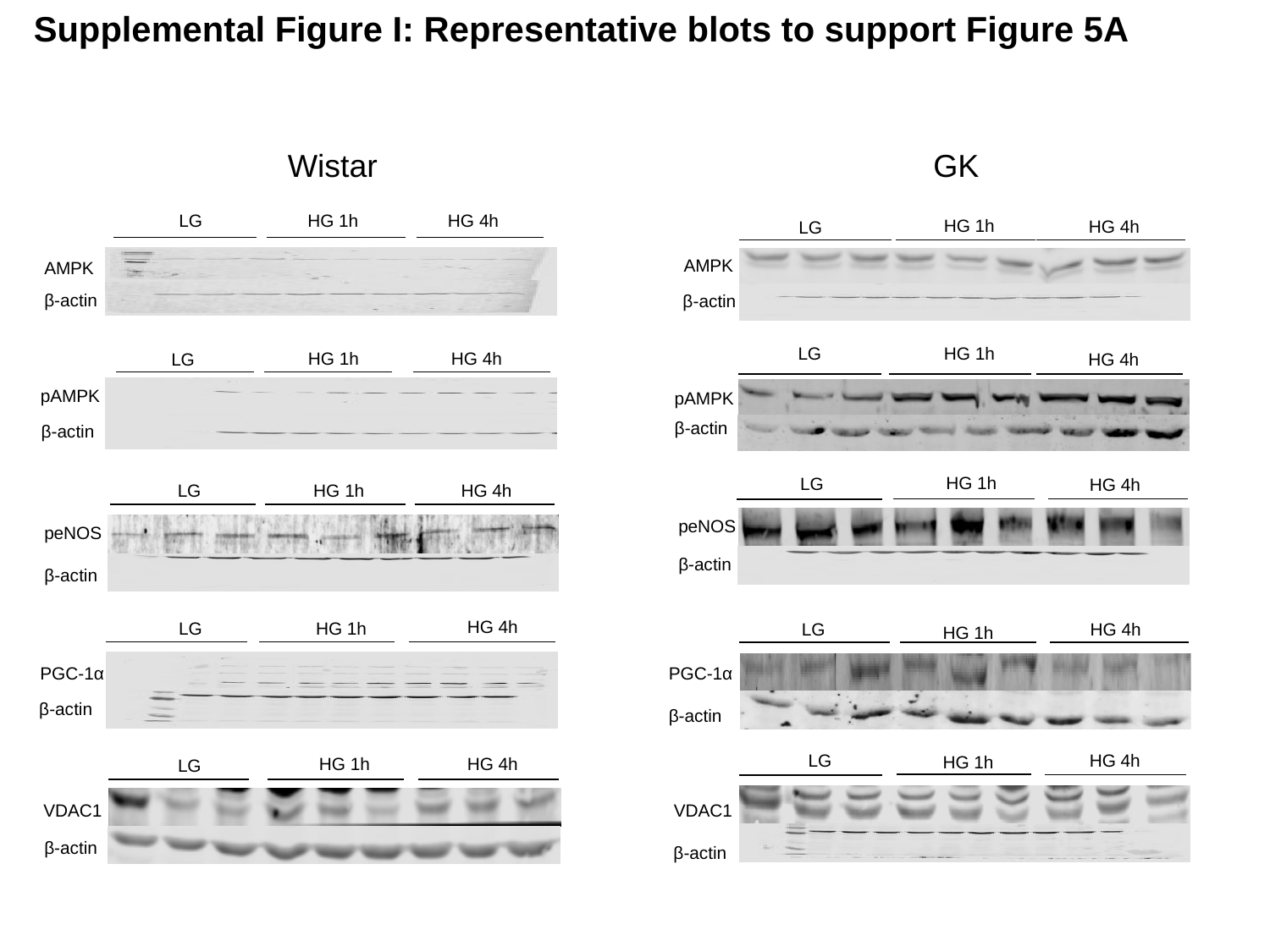

Supplemental Figure I: Representative blots to support Figure 5A
Wistar
GK
HG 1h
LG
AMPK
β-actin
HG 4h
HG 1h
HG 4h
LG
 AMPK
 β-actin
HG 1h
LG
HG 4h
pAMPK
β-actin
 HG 1h
 HG 4h
LG
pAMPK
β-actin
HG 1h
LG
HG 4h
peNOS
β-actin
HG 4h
HG 1h
LG
peNOS
β-actin
HG 4h
LG
 HG 1h
PGC-1α
 β-actin
LG
HG 4h
HG 1h
PGC-1α
β-actin
LG
HG 4h
HG 1h
VDAC1
 β-actin
HG 1h
HG 4h
 LG
 VDAC1
 β-actin

## Slide 2
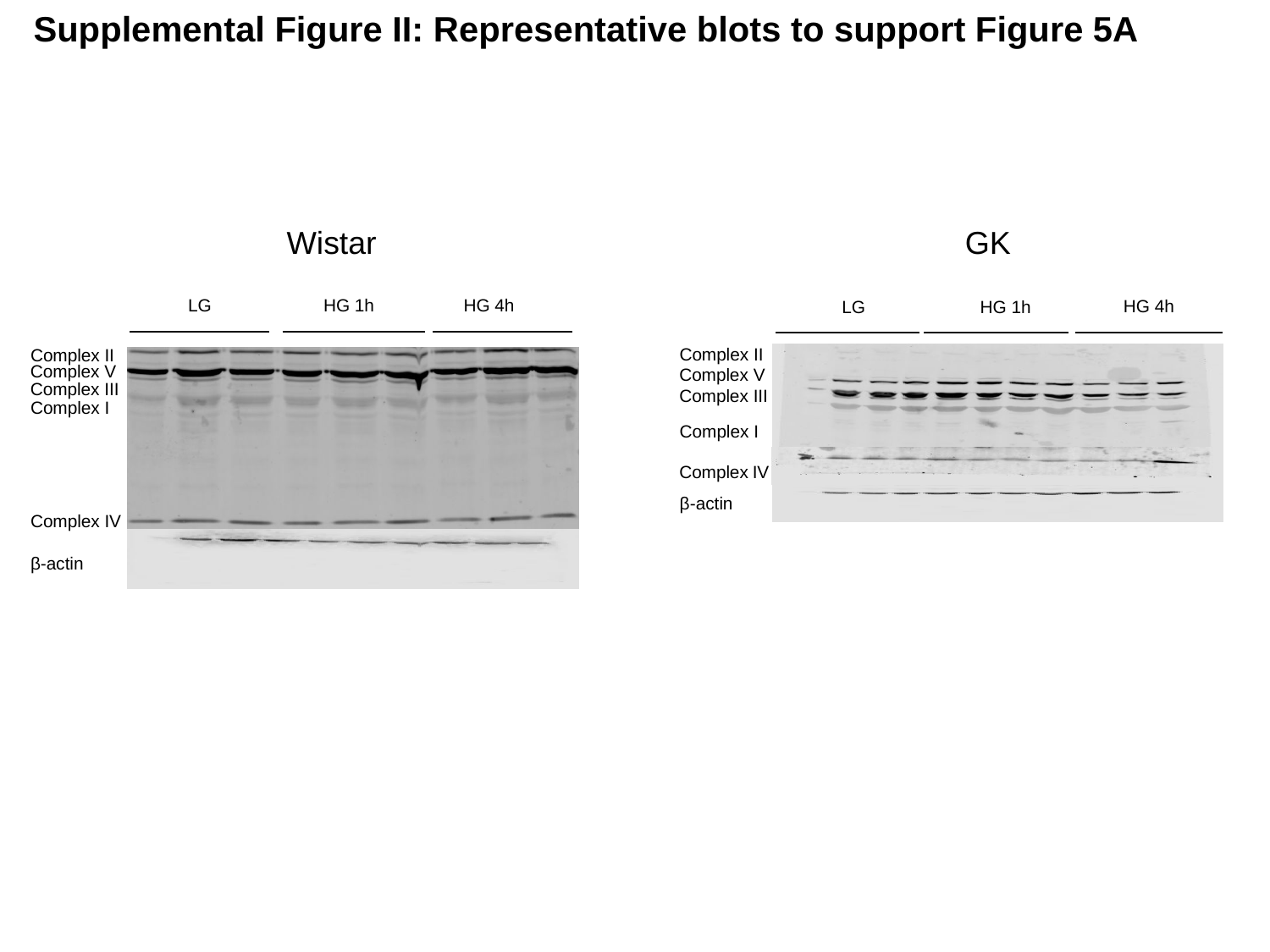

Supplemental Figure II: Representative blots to support Figure 5A
Wistar
GK
HG 4h
LG
HG 1h
Complex II
Complex I
Complex V
Complex III
Complex IV
β-actin
HG 4h
LG
HG 1h
Complex II
Complex V
Complex III
Complex I
Complex IV
β-actin

## Slide 3
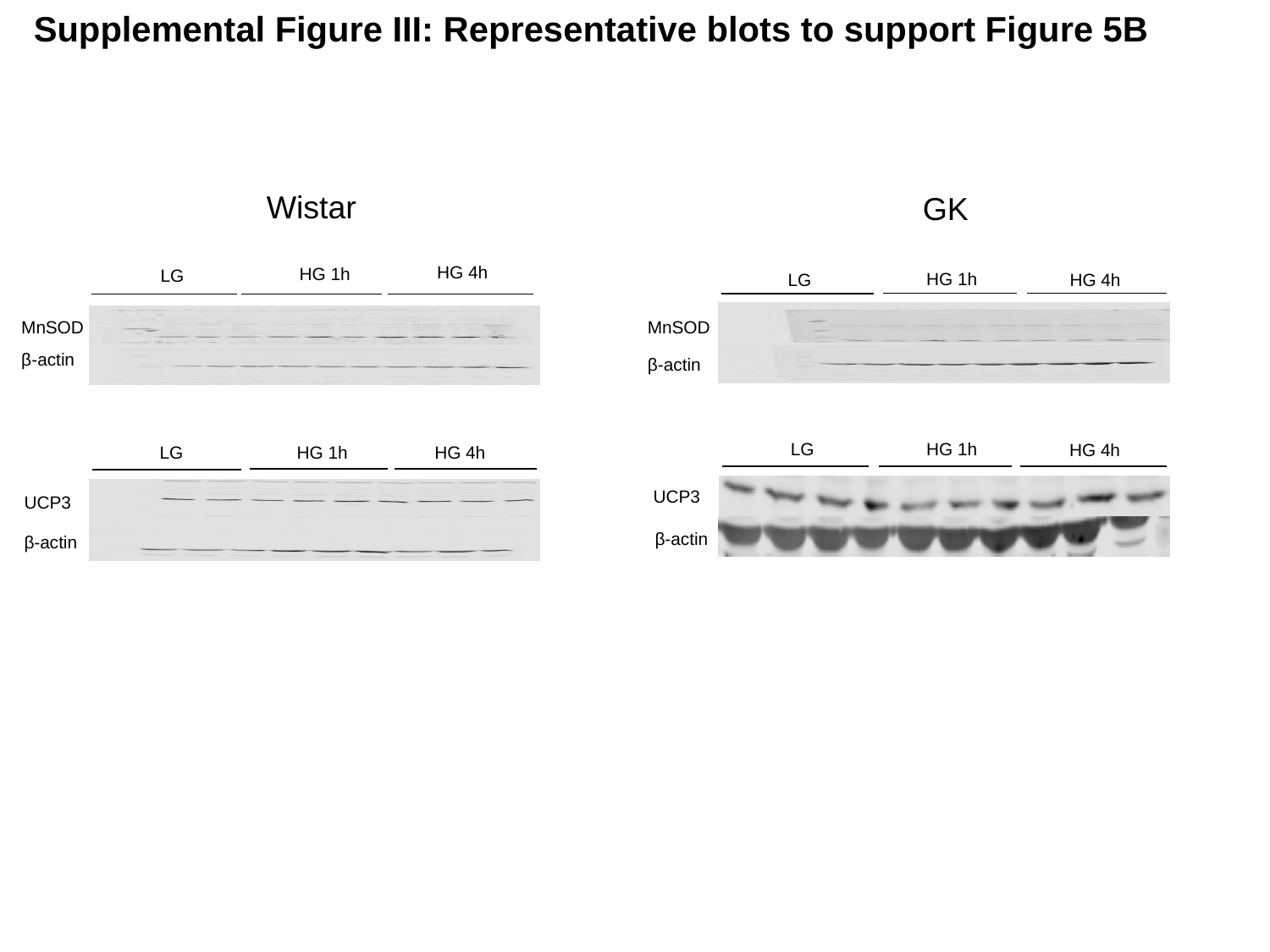

Supplemental Figure III: Representative blots to support Figure 5B
Wistar
GK
 HG 4h
 HG 1h
 LG
MnSOD
β-actin
HG 1h
LG
HG 4h
MnSOD
β-actin
LG
HG 1h
HG 4h
UCP3
β-actin
LG
HG 1h
HG 4h
UCP3
β-actin

## Slide 4
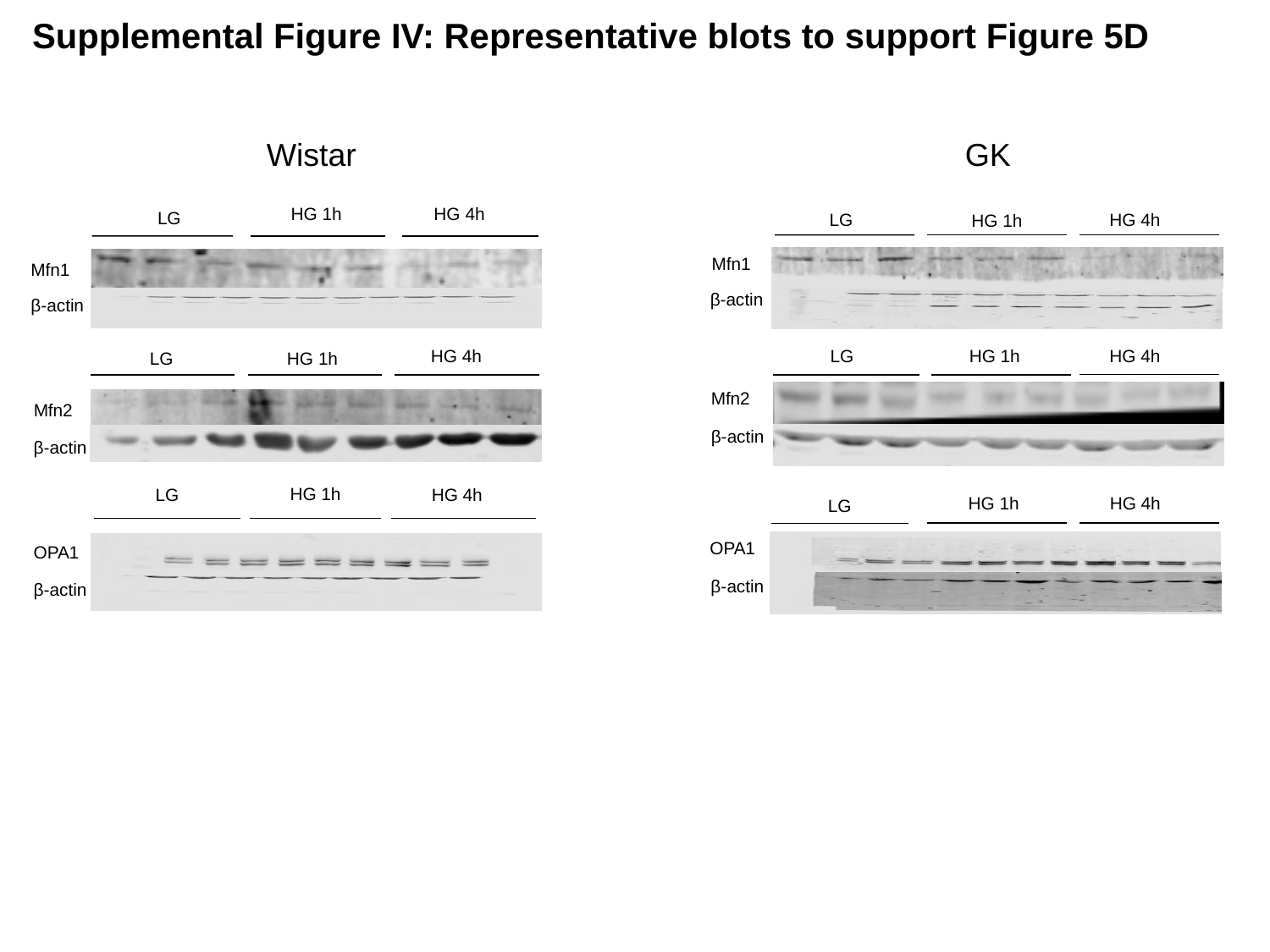

Supplemental Figure IV: Representative blots to support Figure 5D
Wistar
GK
HG 4h
HG 1h
LG
 Mfn1
 β-actin
LG
HG 4h
HG 1h
 Mfn1
 β-actin
HG 4h
LG
HG 1h
Mfn2
β-actin
HG 4h
HG 1h
LG
Mfn2
β-actin
HG 1h
LG
HG 4h
OPA1
β-actin
HG 4h
 HG 1h
 LG
 OPA1
 β-actin

## Slide 5
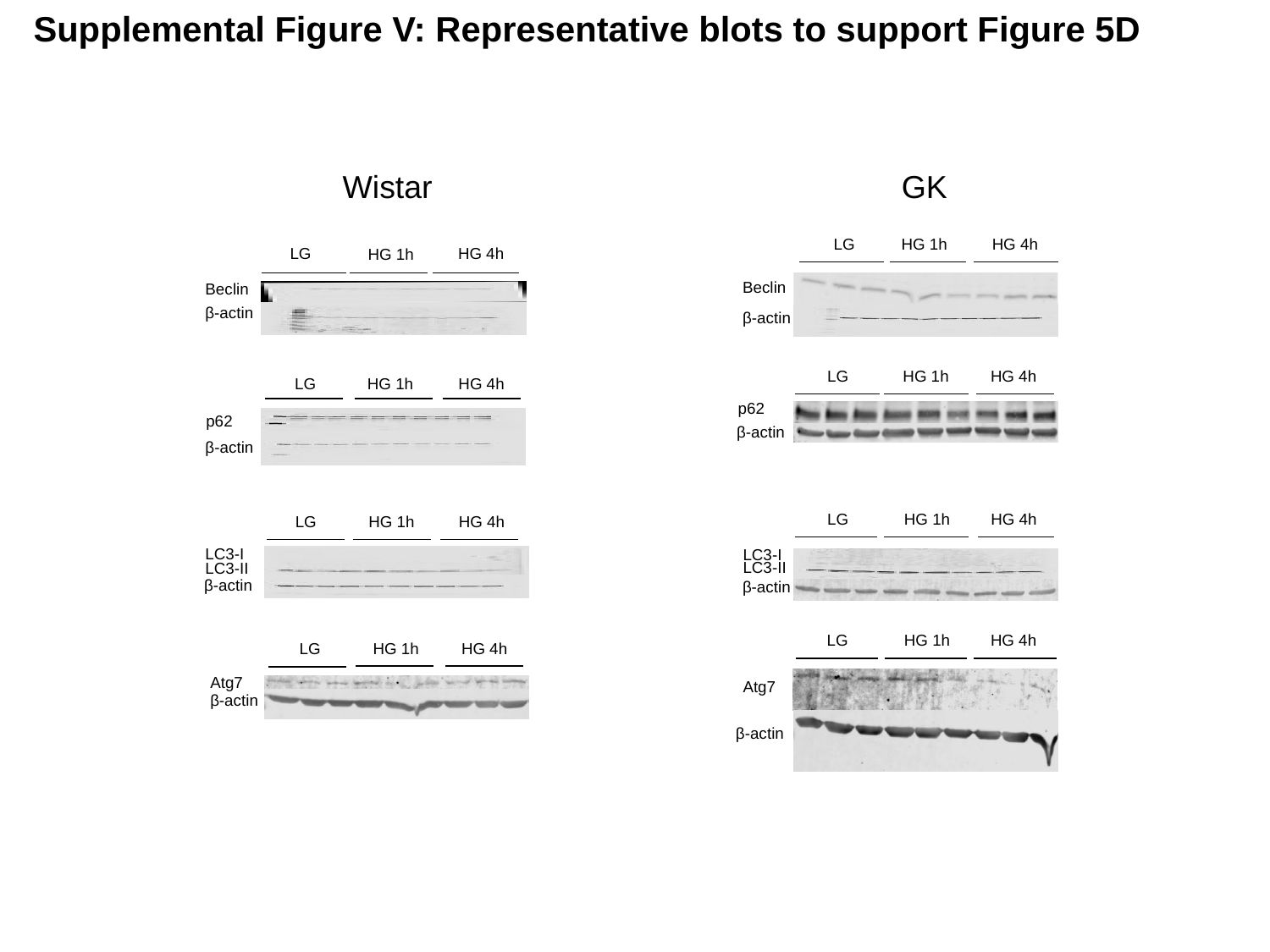

Supplemental Figure V: Representative blots to support Figure 5D
Wistar
GK
LG
HG 1h
HG 4h
Beclin
β-actin
LG
HG 4h
HG 1h
Beclin
β-actin
LG
HG 1h
HG 4h
p62
β-actin
HG 1h
HG 4h
p62
β-actin
LG
LG
HG 1h
HG 4h
LC3-I
β-actin
LC3-II
LG
HG 1h
HG 4h
LC3-I
LC3-II
β-actin
LG
HG 1h
HG 4h
β-actin
Atg7
LG
HG 1h
HG 4h
Atg7
β-actin
